# Family Caregivers Who Would Be Unwilling to Provide Care at the End of Life Again: Findings from the Health Survey for England Population Survey

**DOI:** 10.1371/journal.pone.0146960

**Published:** 2016-01-25

**Authors:** Miriam J. Johnson, Victoria Allgar, Una Macleod, Annie Jones, Steven Oliver, David Currow

**Affiliations:** 1 Hull York Medical School, University of Hull, Hull, United Kingdom; 2 Hull York Medical School, University of York, York, United Kingdom; 3 Discipline, Palliative and Supportive Services, Flinders University, Adelaide, Australia; University of Exeter, UNITED KINGDOM

## Abstract

**Background:**

Family caregivers provide significant care at the end of life. We aimed to describe caregiver characteristics, and of those unwilling to repeat this role under the same circumstances.

**Methods:**

Observational study of adults in private households (Health Survey for England [HSE]). Caregiving questions included: whether someone close to them died within past 5 years; relationship to the deceased; provision, intensity and duration of care; supportive/palliative care services used; willingness to care again; able to carry on with life. Comparison between those willing to care again or not used univariable analyses and an exploratory multiple logistic regression. A descriptive comparison with Health Omnibus Survey (Australia) data was conducted.

**Findings:**

HSE response was 64%. 2167/8861 (25%) respondents had someone close to them die in the previous 5 years. Some level of personal care was provided by 645/8861 (7.3%). 57/632 (9%) former caregivers would be unwilling to provide care again irrespective of time since the death, duration of care, education and income. Younger age (≤65; odds ratio [OR] 2.79; 95% CI 136, 5.74) and use of palliative care services (odds ratio: 1.95, 95% CI: 1.09, 3.48) showed greater willingness to provide care again. Apart from use of palliative care services, findings were remarkably similar to the Australian data.

**Conclusions:**

A significant group of caregivers would be unwilling to provide care again. Older people and those who had *not* used palliative care services were more likely to be unwilling to care again. Barriers preventing access for disadvantaged groups need to be overcome.

## Introduction

The most important predictors of home death are the presence of a family (or friend) caregiver in the home, and both the dying person and caregiver wishing the person to die at home.[1–4] However, the impact on the home space and “normal life” is considerable for family members providing the care; for some, home is transformed into a hospital ward and ‘home’ ceases to be a sanctuary.[4–6]

It is hard to identify the numbers of those providing care for someone at the end of life, partly because of the difficulty in defining when “end of life” begins, and partly because many may be unknown to health services despite initiatives such as caregivers’ registers in UK primary care. However, an estimated 500,000 of those 6.4 million adults in the UK[7, 8] who care for a sick, disabled or elderly person, representing an estimated cost of £119 billion annually[9], provide care for someone at the end of life.[6]In 2011, the South Australian Health Omnibus Survey (HOS) provided a population based prevalence of caregivers providing *hands-on* end-of-life care in the past five years of 10%[10]. This included a network of family and friends.[11] The 2009 to 2010 Allegheny County, PA Behavioral Risk Factor Surveillance System Health Survey found that 7.8% of telephone survey respondents had arranged or provided care for a close friend or family member who had died in the previous year.[12]

Caregiving by intensity of care provided at the *end of life* has been categorized in Australia, (none, rare, intermittent and day-to-day) but not in the UK.[13] The extensive needs of caregivers are summarized in two systematic reviews encompassing 123 quantitative,[14] and 105 qualitative, papers[15]. Needs range from practical help, information and communication, emotional and psychological support to financial and legal issues. Negative effects of caregiving may persist long after the person’s death.[16, 17] However, tools[18] with which to measure benefit and burdens are less well defined. Supportive interventions appear to be effective in reducing caregivers’ psychological distress.[19]

Given predicted rises in those requiring care, health and social care systems will continue to be dependent on unpaid caregivers,[10, 20] it is vital that we understand unpaid caregivers’ characteristics and target support so the role is rewarding, and one they would be willing to take on again if necessary. People will have the opportunity to provide care for someone with terminal illness more than once; for example, even those who cared for a spouse may subsequently be called upon to provide care for another e.g. sibling. Given this likelihood, it is important to understand current care provision and support for caregivers, during care-giving, and into bereavement.[6, 21–23]

The HOS described the characteristics of family and friends who provided care for people close to them at the end of life; 28.3% surveyed had someone close to them die within the last 5 years, and 10% had provided hands-on care.[10] Of this 10% of people, 7.4% would be unwilling to and 16.5% would only “probably” provide care *under the same circumstances*. Unwillingness was more likely in older people and those with a lower education level. “Willingness to care again” may be seen as a proxy measure of the experience of caring for the dying and potentially encompasses clinical care and social support for the patient, and interventions to support the caregiver. Given the population prevalence of one in ten people providing end of life care over a five year period, and the likelihood of the need to do this more than once in a lifetime, a loss of 7.4% of caregivers each time has significant implications for care provision. This current study aimed to describe the characteristics of those in England who had cared for someone who had died, and of those unwilling to repeat this role. The null hypothesis was that there were no socio-demographic factors that predicted unwillingness to care again. In order to provide comparisons of experience between two different national models of health and social care service provision, the caregiver question set used in the HOS was adapted, field tested and included into the Health Survey for England (HSE), 2013.

## Methods

### Health Survey for England questions

#### Adaptation of the Australian question set

As this question set had been extensively field tested in a developed English-speaking nation with national health and social services similar to England, an in-depth cognitive testing process was deemed unnecessary by the HSE team. However, in order to test the acceptability and face/content validity of the questions in England, two focus groups of a convenience sample of the general public were conducted; one in an urban East Yorkshire and one in rural North Yorkshire. Participants provided written informed consent to participate in the focus groups. Institutional ethical approval for the focus groups was provided by the Hull York Medical School Ethics Committee, including approval for the consent procedure.

Particular attention was paid to the phrasing and meaning of the questions. The rural group included those with and without experience of caring for people at the end of life.

Any changes were agreed by consensus during the focus groups, then by the research team and finally, approved by the HSE team. The questions were also reviewed by the Department of Health End of Life lead and an independent researcher in the field (acknowledgements).

The subsequent revised set (S1 Table) was further refined following pilot testing by the HSE team and following a few weeks’ of survey activity.

#### Survey method: Health Survey for England

The Health Survey for England (HSE) is commissioned by the NHS Information Centre for Health and Social Care for the Department of Health. Since 1994, this annual survey has been carried out in collaboration with the Health and Social Survey Research group at the Department of Epidemiology at University College London. Health and health related behaviours in adults and children are surveyed using a visit from a trained interviewer and a nurse. Annual core elements include socio-demographic data at the household and respondent level. Researchers may submit their own question module. Anonymised demographic data and the responses to the researchers’ own submitted questions are provided. A random probability sample of households (9,408 addresses in 588 postcode sectors) was surveyed. Adults (age 16 or over) were interviewed at households identified at the selected addresses. Addresses were issued from January to December 2013, and fieldwork was completed in March 2014. Further details can be found in the full report of methods.[24] For the elements involved in this report, verbal consent only was sought. Verbal consent was not recorded assuming that those who took part in the survey, and provided data had consented to do so. In England, minors between the ages of 16–18 are presumed competent to give consent and thus the consent processes are the same as for adults. The HSE included the question set in their ethics approval processes (including consent) for the 2013 survey, obtained from the Oxford A Research Ethics Committee (reference 12/SC/0317).

#### Sample size

In the HOS, 9.5% of the sample was identified as ‘having provided hands-on care for someone close to them who had died in the last 5 years’. From the HSE survey (sample size >8000), we therefore expected data on at least 800 respondents allowing estimation of proportions to within +/-3.5%, based on 95% confidence.

### Statistical Analysis

The main analysis was a comparison of caregivers who would/probably would take on the caregiving role again and those who probably would/would not. The analysis plan followed that conducted for the HOS data[10] to allow comparison. The data were weighted in line with HSE weights for individuals to help account for non-response bias.[24] Mean (SD) and n (%) were used to describe the demographic characteristics of the caregivers, care giving characteristics, service use and place of death of the deceased for each group. Univariate analysis was undertaken to compare the groups using a Chi-square test for categorical data and a t-test for continuous data e.g. age. A p-value <0.05 was considered to indicate statistical significance. No adjustments were made for multiple significance testing.[25], [26] Missing data were not imputed.

An exploratory logistic regression model was created from the most significant factors from the univariate analysis and plausible factors from the literature.[2, 10] An estimated 10 cases are required for each single degree of freedom predictor (including intercept);[27] 57 respondents expressing unwillingness to care would allow a logistic regression model with up to five predictors. Caregiver characteristics that may change as a result of the death, such as place of residence, work status, and household income were excluded. The month and year of death was provided, but not the interview date so time from death to interview could not be calculated. The year of death was used to explore the relationship between time since death and willingness to care. Analysis was undertaken on SPSS (Released 2013. IBM SPSS Statistics for Windows, Version 22.0. Armonk, NY: IBM Corp).

## Results

### Descriptive Data

A household response rate of 64% was achieved for the HSE survey 2013. Respondents were representative of the Annual Mid-year Population Estimates, 2013 in terms of age and sex.[28] Overall, 2167/8861 (25%) respondents had someone close to them who had died in the previous 5 years. Information about the personal care provided was available for 2163 (3 declined to answer, 2 “don’t know”). Daily care was provided by 307/2163 (14%) caregivers, 252 (12%) caregivers provided occasional/intermittent care, and 86 (4%) rarely. Some level of personal care was provided by 645 (645/8861; 7.3%) respondents.

#### Willingness to care again

Willingness to care again was defined as “definitely/would probably” provide care again under the same circumstances. 632/645 caregivers completed the question about being willing to provide care again, of whom 575 (91%) would be willing to provide care again and 57 (9%) would “probably not/would not”.

The characteristics of people who would and who would not be willing to provide care again are shown in Table 1.

**Table 1:** Characteristics of caregivers who would or would not be willing to provide care again.

	Would not take on the caregiving role again (n = 57)	Would take on the caregiving role again (n = 575)	p-value
**Factors that do not change as care giving in relinquished**			
**Age**			
Mean (sd)	63.3 (18.1)	48.5 (17.7)	**<0.001**
Aged ≥65	30 (53%)	114 (20%)	**<0.001**
**Gender** (Male)	21 (63%)	225 (39%)	0.735
**Education**		n = 573	<0.**001**
No Qualifications	23 (40%)	111 (19%)	
Below degree	29 (51%)	299 (52%)	
NVQ4/NVQ5/Degree or equivalent	5 (9%)	163 (28%)	
**Relationship to deceased (Spouse)**	22 (38%)	76 (13%)	**<0.001**
**Factors that may change as caregiving in relinquished**			
**Household Income Quintiles**	n = 43	n = 454	**0.002**
< = £12,803	11 (26%)	82 (18%)	
>£12803 < = £19,500	18 (42%)	88 (19%)	
>£19,500 < = £29,865	5 (12%)	96 (21%)	
>£29,865 < = £49,016	7 (16%)	105 (23%)	
>£49,016	2 (5%)	83 (18%)	
**Working Status (In work)**	15 (26%)	352 (61%)	**<0.001**
**Caregiving characteristics**			
**Level of care (Daily)**		n = 566	**0.004**
	39 (68%)	275 (49%)	
**Length of care (≤one year)**	34 (60%)	387 (67%)	0.242
**Palliative care used**	27/56 (48%)	371/569 (65%)	**0.012**
**Reason palliative care not used (Multiple response)**	29	198	
The service was not available	2 (7%)	15 (8%)	0.897
Didn’t know about palliative care services	4 (14%)	12 (6%)	0.129
Service was not wanted	10 (35%)	27 (14%)	0.005
Family/friends looked after person	5 (17%)	28 (14%)	0.714
Death was sudden	5 (17%)	50 (25%)	0.347
Died in hospital	8 (28%)	75 (38%)	0.282
Other reasons	4 (13%)	7 (4%)	0.020
Died in another country	0 (0%)	1 (0.5%)	0.701
**Had special help given**	20 (35%)	194/573 (34%)	0.852
**Personal care only**	37 (65%)	381/567 (67%)	0.727
**Year person died**			0.335
2008	11 (19%)	61 (11%)	
2009	8 (14%)	91 (16%)	
2010	8 (14%)	116 (20%)	
2011	13 (23%)	119 (21%)	
2012	9 (16%)	122 (21%)	
2013	8 (14%)	65 (11%)	
**Post care factors**			
Continue with my life –		n = 576	**0.020**
Able to continue	42 (74%)	488 (85%)	
Starting to continue	12 (21%)	81 (14%)	
Not been able to continue	3 (5%)	7 (1%)	
**The deceased**			
Diagnosis (Cancer)	35 (61%)	408 (71%)	0.133
Place of death (Hospital or Hospice)	26 (46%)	268 (47%)	0.886

#### Demographic characteristics

The caregivers *un*willing to care again were older ((63.3 years (SD 18.1) than those who were willing (48.5 (SD 17.7), p<0.001). Fewer caregivers ≥ 65 years were willing to care again (20%) than those who would not (53%; p<0.001).

Of the people willing to care again, 28% had National Vocational Qualification (NVQ)4/NVQ5/Degree or equivalent, whereas of those who were not, 9% had NVQ4/NVQ5/Degree or equivalent (p<0.001).

We dichotomised the relationship with the deceased between spouses (16%) and “others” (parent [34%], child [4%], sibling [6%], other relative [31%], friends [8%], and others [2%]). Spousal relationship was significant; of those willing to care again, 13% had cared for a spouse, whereas of those who were unwilling, 38% had cared for a spouse (p<0.001).

Lower household income was associated with a decreased likelihood of taking on the caring role again (p = 0.002). Of people willing to care again, 18% had a lower quintile household income and 18% had a highest quintile household income, compared with 26% and 5% respectively for those unwilling.

#### Caregiving experience

Those providing more intense care were less likely to be willing to care again; 49% providing daily care would care again compared with 68% who would not (p = 0.004).

More people who would care again had used a specialist palliative care service (65%), than those who were unwilling (48%, p = 0.012). Where palliative services were not used a variety of reasons were given; most commonly that the person died in hospital (36%), the death was sudden (24%), the service was not wanted (17%) and family and friends provided the care (14%). However, 7% cited unavailability and 7% did not know about the services. Fewer caregivers willing to care again stated that a palliative care service was unwanted (14%) than those who would be unwilling (35%; p = 0.005).

If the person had died of cancer, they were more likely to have received specialist palliative care (75% cancer; 36% non-cancer). If the person had died of cancer, of those who said they would care again, 77% of decedents had received specialist palliative care, compared with 51% of those whose caregivers would be unwilling to care again (p = 0.001). This pattern was not seen in those where the deceased had a non-malignant disease (36% willing; 41% unwilling; p = 0.623).

Of the people who said they would care again, 85% had been able to “continue with life”, whereas of those who were unwilling, 74% had been able to “continue with life” (p = 0.020).

There was no relationship between time elapsed since the death and willingness to care again. The proportions of those unwilling to care again did not change: 2008 (15%); 2009 (8%); 2010 (7%); 2011 (10%); 2012 (7%); 2013 (11%). p = 0.335.

#### Logistic regression model

Given the small number of people who indicated that they would be unwilling to provide care again (n = 57), the regression analysis used willingness to provide care again as the dependent variable with caregiver age, highest level of education, use of palliative care services and spousal status as the independent variables. Two significant factors helped to explain willingness to care again: younger age (≤65; odds ratio [OR] 2.79; 95% CI 136, 5.74) and use of palliative care services (odds ratio: 1.95, 95% CI: 1.09, 3.48). For this analysis, the Hosmer and Lemeshow goodness of fit (p = 0.538) suggested that the model adequately fits the data, and the Omnibus Tests of Model coefficients (p< 0.001) confirmed this. The Nagelkerke R-square was 0.158.

#### Sensitivity analysis

In view of the likely complex relationship between age and spousal status, we conducted a sensitivity analysis to control for who the caregiver cared for; spouse, child, parent, sibling, friend or other relative. This model showed that who they cared for was not significant (p = 0.156) but age (p = 0.027) and palliative care (p = 0.016) were.

## Discussion

Age of caregiver and use of palliative care services independently predicted willingness to care again. Older caregivers and where decedents did not use palliative care services were less likely to be willing to be caregivers again under the same circumstances. Given the challenges of identifying family caregivers who have provided care for the dying, and of delineating representative groups who have and who have not used palliative care services, this is one of few studies to demonstrate benefit (willingness to care again) from palliative care services after caregivers’ roles are completed,[2, 12, 29, 30] and is consistent with Seaman JB and colleagues who found that involvement of hospice services improved end of life quality outcomes and increased caregiver involvement in care.[12] In this HSE study, there was a significant association between “continuing with life” and willingness to care again. Difficulty in being able to continue with life may indicate more complex grief. It is possible that involvement with palliative care services might be helpful in this regard, but our data do not allow more than conjecture, and this variable did not remain in the final model.

Less use of palliative care services by older patients has been reported previously.[31, 32] Although the age of the patient and caregiver may not be same, they do correlate moderately. However, caregiver and patient age are independent in some situations; age of caregiver is an independent predictor for use of palliative care services e.g. younger caregivers are more likely to use home palliative care nursing services although it is uncertain whether this is because of greater effectiveness at accessing support, or greater needs.[32] Our data suggest that less use by older caregivers is not because of fewer needs. As older caregivers are more likely to have age-related morbidities and disability, this is unsurprising. Either these needs are not being met or there are other factors at play e.g. variability in palliative care service delivery, poor understanding about what services could offer or how to access them. Palliative care use was more likely for those dying of cancer in which situation use was significantly associated with caregivers’ willingness to care again. This may be due to a variety of factors, but poorer use of palliative care services for people with non-malignant disease is an ongoing international issue.[33–35] The barriers to palliative care access for people with non-malignant disease are well described and include the different disease trajectories whereby palliative care needs are poorly recognised.[36–38]

Interestingly, involvement of other care services (for example social services, a private care company, meals on wheels, voluntary groups) did not appear to be an influencing factor and neither was duration, unlike intensity, of care.[10] Place of death did not affect caregivers’ willingness to care again. A study of factors in relation to a good death, looking at the views of patients, caregivers and professionals showed that caregivers considered dying at home to be important, more so than patients.[39] However, concerns such as symptom control, dignity, access to family, trust in the healthcare team were ranked above place of death by both patients and caregivers. Hence palliative care services may be providing a process of support tailored both to need and family routine (credible skilled information, education and training about future care needs, medication and symptom managements and contingency planning)[40] for those with complex needs which, if addressed, would have a bigger direct impact for the caregiver than general support. The challenge is to identify those with increased need and triage resources. Despite policy statements[41, 42] and efforts to develop and test interventions to support caregivers, there is no consensus regarding the most effective approach. Therefore, honing the use of current resources is essential.

### Strengths and limitations

This population based survey used robust sampling methodology and questions adapted to ensure cultural competence whilst maintaining content for comparison with findings from a country with a different health and social care service delivery model.

However, the age of the deceased is unknown and we do not have data on the models of palliative care services allowing more in depth comment.

This first use of such a question set in the HSE only addressed issued to do with activities of daily living and did not address some of the medical and pain management issues which are increasingly challenging as people are terminally ill.

### Similarities and differences to Health Omnibus Survey South Australia

In keeping with similar population age and mortality patterns, the results of these two population based surveys show similar proportions of people who had someone close to them die in the last 5 years (28.3% Australia vs 25% England). However, although Australian and UK health and social care services have similarities, there are differences. Despite these there were strikingly similar proportions of those who provided personal care (9.5% vs 7.3%), and were unwilling to care again (7% vs 9% caregivers).[10] It appears that care services are sufficient for most people, delivering marginal benefit for many: most cope with most things most of the time. Both surveys identify increasing age as an associated factor although use of palliative care services was not an explanatory variable in Australia, and lower educational level was not an explanatory variable in England.

### Implications for practice, policy and research

In this important cross-cultural confirmatory study, it is reassuring that most people would be prepared to care again. However, there are important lessons and identify important targets for improved care, both for those who are likely to be asked to care again, and for those who will care for the first, and possibly only, time but who are at high risk of having a less supported experience. The inequity of access to palliative care services for people with non-malignant disease is well known; in practice many of those with non-malignant conditions are older. There is an urgent need to reduce barriers to disadvantaged groups in accessing palliative care services. Some progress has been made, but palliative care services remain primarily for people with cancer.[43] Morris and colleagues make suggestions for family caregiver support: education and training in medication management and symptom control; recognition that caregivers have needs in their own right rather than solely viewing as “co-workers”; respect for family routines and plan professional care interaction around their timetable[6]. These considerations are possible within current resources, although require changing attitudes to provide thoughtful, family-centred configuration of services.

Given the demographic and economic circumstances changes occurring in many places globally, it is crucial that caregivers are supported, recognising that “one size” does not fit everybody.

“Willingness to care again under the same circumstances” is a broad measure of experience. Further work is needed to investigate whether stated willingness predicts future behaviour and to what extent willingness reflects an overall acceptable experience and adequate support, or actions driven by major personal factors. Now it has been shown that a caregiver question set is possible in this context in the UK, further question sets administered through the HSE can include questions about other important aspects of caregiving.

## Conclusions

Most people who have provided end of life care for someone close to them would be prepared to provide care again. However, a significant group would not. Younger age of caregiver and use of palliative care services were associated with willingness to care again highlighting the need for appropriately skilled support for patient and their families while in this role and subsequently. Barriers preventing access for disadvantaged groups need to be actively overcome.

## Supporting Information

S1 Table Adaptation of Australian Question Set. (PDF)
