# Supplementary material for: Family Caregivers Who Would Be Unwilling to Provide Care at the End of Life Again: Findings from the Health Survey for England Population Survey
Source: PLoS One. 2016 Jan 25;11(1):e0146960. doi: 10.1371/journal.pone.0146960 (PMC4726543; doi:10.1371/journal.pone.0146960)
Supplement: S1 Table — (PDF) [file pone.0146960.s001.pdf]

S1 **Table. Adaptation of caregiver question set**

**Original**	**Modified Question after focus groups**	**After field testing and team discussion**
1. **In the past five years, has anyone close to you died of a terminal illness like cancer, motor neurone disease, or emphysema? If yes, could you say what that illness was?** (Interviewer: If more than one person, ask about the most recent person to die of a terminal illness.)	**In the past five years, has anyone close to you died of a terminal illness? If yes, could you say what that illness was?** (Interviewer: If more than one person, ask about the most recent person to die of a terminal illness.)	**In the past five years, has anyone close to you died of a terminal illness like cancer, motor neurone disease, or emphysema? If yes, could you say what that illness was?** (Interviewer: If more than one person, ask about the most recent person to die of a terminal illness.)
❑ No<br>❑ Emphysema/other lung disease<br>❑ End stage heart failure<br>❑ End stage liver failure<br>❑ Cancer<br>❑ Motor neurone disease/multiple sclerosis<br>❑ End stage kidney failure<br>❑ HIV/AIDS<br>❑ Other (specify)	Options – no change	Options – no change

❑ Don't know illness		
**2. How long ago did this person die?**<br>_____ years _____ months	**When did this person die?**<br>_____ month _____ year<br><br>**Where did they die?**<br>❑ Home (theirs)<br>❑ Home (yours)<br>❑ Home (we lived together)<br>❑ hospital<br>❑ hospice<br>❑ nursing care home<br>❑ residential care home<br>❑ Other (specify)	This is now two separate questions (when and where)<br><br>Show card for options
3. **What was your relationship to this person?** (Interviewer: If more than one person, ask about the most recent person to die of a terminal illness.)<br><br>❑ Spouse/partner<br>❑ Parent<br>❑ Child	**What was your relationship to this person?**<br><br>**This person was my....**<br>❑ Spouse/partner<br>❑ Parent<br>❑ Child	**What was your relationship to this person?**<br>INTERVIEWER: If more than one person, ask about the person who died most recently.<br><br>Options – no change

❑ Sibling<br>❑ Other relative<br>❑ Friend<br>❑ Other (specify)	❑ Brother/sister (including half or step brother or sister)<br>❑ Other relative<br>❑ Friend<br>❑ Other (specify)	
4. **What was your most involved level of care for this person?**<br>*[Put responses on show card]*<br>❑ Day-to-day hands on care<br>❑ Intermittent hands on care<br>❑ Rare hands on care<br>❑ Didn't provide any care but they were still close to me	**What was your maximum level of personal care for this individual? Tick one, and please estimate how long this was (circle time).**<br>*[Put responses on show card]*<br>❑ Daily:<br>○ Days<br>○ Weeks<br>○ Months<br>○ more than a year<br>❑ Occasional : ______<br>○ Days<br>○ Weeks<br>○ Months<br>○ more than a year<br>❑ Rare:	**What was your maximum level of personal care for this individual?**<br>INTERVIEWER: By personal care I mean things like helping with washing, dressing, going to the toilet, or eating.<br>Show card for options<br>❑ Daily ______<br>❑ Occasional/intermittent<br>❑ Rare ______<br>❑ Didn't provide any personal care but they were still close to me<br>❑ Other (PLEASE SPECIFY)

		○ Days<br>○ Weeks<br>○ Months<br>○ more than a year<br><br>❑ Didn't provide any personal care but they were still close to me<br><br>❑ Other (specify): __<br>○ Days<br>○ Weeks<br>○ Months<br>○ more than a year	INTERVIEWER: Thinking about the time when you were providing help like this, how long was this for?<br><br>❑ Days<br>❑ Weeks<br>❑ Months<br>❑ More than a year
No comparator question this is replaced by an additional question which now the new question shown in the columns to the right<br>5. **Think back to when they were first diagnosed. Was the period after their diagnosis through to and including their death better or worse than you would have expected?**		**Did you look after or give special help to them in any other way? If yes, what was your most maximum level of help for this person?** (Interviewer: to have list of examples: company, errands, laundry, shopping, giving lifts, taking to appointments or out for recreation etc)<br>*[Put responses on show card]*	**Did you look after or give special help to them in any other way? If yes, what was your most maximum level of help for this person?** (Interviewer: to have list of examples: company, errands, laundry, shopping, giving lifts, taking to appointments or out for recreation etc)

	[Put responses on show card]<br><br>❑ Much better than expected<br>❑ Better than expected<br>❑ As expected<br>❑ Worse than expected<br>❑ Much worse than expected<br>❑ I didn't know what to expect	❑ Daily:<br>○ Days<br>○ Weeks<br>○ Months<br>○ more than a year<br><br>❑ Occasional : ____<br>○ Days<br>○ Weeks<br>○ Months<br>○ more than a year<br><br>❑ Rare:<br>○ Days<br>○ Weeks<br>○ Months<br>○ more than a year<br><br>❑ Didn't provide any personal care but they were still close to me<br><br>❑ Other (specify): __	Show card for options<br><br>❑ Daily _______<br>❑ Occasional/intermittent<br>❑ Rare _______<br>❑ Didn't provide any personal care but they were still close to me<br>❑ Other (PLEASE SPECIFY)<br><br>INTERVIEWER: Thinking about the time when you were providing help like this, how long was this for?<br><br>❑ Days<br>❑ Weeks<br>❑ Months<br>❑ More than a year

		○ Days<br>○ Weeks<br>○ Months<br>○ more than a year	
**6.**	**Did this person who died of a terminal illness use a palliative care service?** (Examiner: "A palliative care service aims to comfort, not to cure, to relieve pain and distress for people who are dying and to support patients, families and friends in approaching death and coping with grief.")<br>❑ Yes<br>❑ No<br>❑ Don't know	**Was a palliative care service used?** (Interviewer: "A palliative care service aims to comfort, not to cure, to relieve pain and distress for people who are dying and to support patients, families and friends in approaching death and coping with grief. Typical services include; hospice, visits from Marie Curie or/and Macmillan nurses.")<br>Options – no change<br>**If no, could you please say why a palliative care service was not used?**<br>Options – no change	Show card for options<br>No further change
**7.**	**If no, could you please say why a palliative care service was not used?**		

❑	A service was not available<br>❑ Didn't know about such a service<br>❑ Service was not wanted<br>❑ Family/friends looked after<br>❑ Death was sudden<br>❑ The person died in hospital<br>❑ Other (specify)<br>❑ Don't know		
No	comparator	**Were any other care services used?** (Interviewer: "For example social services, a private care company, meals on wheels, voluntary groups")<br>❑ Yes<br>❑ No<br>❑ Don't know<br><br>**If no, could you please say why other care services were not used?**	Show card for options<br>No further change

		❑ A service was not available<br>❑ Didn't know about such a service<br>❑ Service was not wanted<br>❑ Family/friends looked after<br>❑ Death was sudden<br>❑ The person died in hospital<br>❑ Other (specify)	
**8.**	**Since this person died, have you been able to move on with your life?**<br>*[Put responses on show card]*<br>❑ I have been able to move on with my life<br>❑ I am starting to move on with my life<br>❑ I have not been able to move on with my life	**Since this person died, have you been able to continue with your life?**<br>*[Put responses on show card]*<br>❑ I have been able to continue with my life<br>❑ I am starting to continue with my life<br>❑ I have not been able to continue with my life	Show card for options<br>No further change
**9.**	**Would you take on the role of caring again in similar circumstances?**	**Would you take on the role of caring again in similar circumstances?**	Show card for options<br>No further change

	❑ I would definitely take on the caring role again<br>❑ I would probably take on the caring role again<br>❑ I would not take on the caring role again	❑ I would definitely take on the caring role again<br>❑ I would probably take on the caring role again<br>❑ I would probably not take on the caring role again<br>❑ I would not take on the caring role again	

2

3
